# Stage at Presentation and Clinical Characteristics of Breast Cancer Patients at the Butaro Cancer Centre of Excellence, Rwanda: A Retrospective Chart Review

**DOI:** 10.21203/rs.3.rs-11308254/v1

**Published:** 2026-10-11

**Authors:** Jean Paul Majyambere, Betel Amdeslassie Fenta, Naol Belema Gemechu, Phillip J. Hsu, Anteneh Gadisa Belachew, Anne Niyigena, Alexandra Fehr, Alex Y. Z Huang, Mulugeta Tenna, Yuval Cohen, Nefti Bempong-Ahun, Wendy Williams, Robert Riviello, Abebe Bekele, Geoff Ibbotson, Barnabas Tobi Alayande

**Affiliations:** Butaro Hospital; University of Global Health Equity; University of Global Health Equity; University of Michigan; University of Global Health Equity; University of Global Health Equity; University of Global Health Equity; University of Illinois Chicago; University of Global Health Equity; Global Surgery Foundation; Global Surgery Foundation; Brigham and Women's Hospital; Harvard Medical University; University of Global Health Equity; Global Surgery Foundation; University of Global Health Equity

## Abstract

**Background:**

Breast cancer is the leading cancer among women in sub-Saharan Africa, where late-stage presentation contributes to poor outcomes. Understanding the risk-factor profile, comorbidity burden, and functional status of patients at presentation is essential to guide early-detection and management strategies in resource-limited settings.

**Methods:**

We conducted a retrospective chart review of patients who underwent surgery for breast cancer at the Butaro Cancer Center of Excellence (BCCOE), Rwanda, between 2018 and 2023. From 422 patients identified, 300 charts were selected through simple random sampling and reviewed using a pretested REDCap tool drawing on electronic and paper-based records. Data was analyzed in IBM SPSS Statistics (version 29), and disease was staged according to the AJCC TNM system.

**Results:**

The mean age at presentation was 50.8 years (SD = 11.8; range 28–82); 98% of patients were female, 94% had health insurance, and 74% were farmers. At least one risk factor was present in 65% of patients, with alcohol consumption (55.3%) and tobacco smoking (21.7%) the most common modifiable factors, followed by family history (5.3%) and nulliparity (3.7%). Comorbidities were frequent, affecting 60.3% of patients; hypertension (25.7%) and HIV/AIDS (7.0%) predominated. Most patients had good functional status at diagnosis (ECOG 0, 81.5%; ECOG 1, 16.4%). Of 281 staged patients (19 of 300 lacked complete TNM staging data), Stage III (38.4%) and Stage II (37.0%) were most common, and late-stage disease (Stage III–IV) accounted for 47.3% of cases.

**Conclusion:**

Breast cancer patients at BCCOE are diagnosed at a younger age than the global average and carry a high burden of modifiable risk factors and comorbidities. The substantial proportion presenting at late stage may complicate treatment and compromise survival, emphasizing the need for strengthened awareness and early-detection efforts. By centering on a rural referral hospital, this study adds to the limited literature on breast cancer presentation in Rwanda.

## Introduction

Delays in the continuum of breast cancer care remain a defining challenge in low- and middle-income countries (LMICs), shaping both the stage at diagnosis and the overall clinical profile of patients at presentation (1). These delays, which often span from symptom recognition to health-seeking behavior and eventual initiation of treatment, contribute to a higher burden of advanced disease and compromised functional status at the time of diagnosis. Consequently, patients frequently present with more aggressive disease characteristics, poorer performance status, and limited therapeutic options, all of which adversely affect outcomes (Brand et al., 2019). Understanding the interplay between delays in care and the clinical and sociodemographic characteristics of breast cancer patients is therefore essential to contextualize patterns of presentation and to inform strategies aimed at improving early detection and timely management.

Despite a higher prevalence of breast cancer in developed regions, breast cancer mortality rates are higher in LMICs than in high-income countries (HIC), (2). The high mortality and morbidity rate reported in LMICs can be attributed to several factors, such as late-stage diagnosis, delayed treatment, lack of integrated multimodal treatment modalities, and poorly addressed co-morbidities (3). Literature also points to a shift in the prevalence of breast cancer attributable to changes in modifiable and non-modifiable risk factors in these developing countries (4). Improving the outcomes of breast cancer patients often depends on prevention and early detection of cancer.

Late presentation and diagnosis of breast cancer are significant contributors to poor outcomes and increased mortality. A systematic review of the literature on Sub-Saharan Africa examined the stage at diagnosis and its temporal trends, revealing that 74% of patients presented at a late stage of the disease (5). Although the trend over time has gradually improved, delayed presentation and late diagnosis remain more prevalent than in HICs, further exacerbating disparities in outcomes (Jedy-Agba et al., 2016).

The outcome of treatment and response of patients to several modalities of cancer treatment depends not only on the delay at presentation but also on the status of performance at diagnosis (6). Performance at presentation for oncology patients is affected by sociodemographic factors such as age, nutritional status, and comorbidities. Several tools have been developed to summarize these factors and predict the outcome of cancer patients (7). ECOG performance status is one of such tools routinely used to assess if patients are fit for systemic cancer treatments and predict response (8). A higher ECOG score (ECOG 3 and 4) has been predictive of mortality in breast cancer patients, and patients with an ECOG performance status of 3 or higher experienced a greater likelihood of in-hospital mortality, independent of their age or stage of disease (8, 9).

Improving survival for breast cancer patients therefore depends on stage at presentation. In this study, we examined the presentation of breast cancer in Rwanda through a retrospective chart review of a rural cancer center that treats cancer patients from all over Rwanda. The study presented the stage of presentation of breast cancer patients, among other factors in presentation, such as risk factors and other comorbidities that might affect treatment outcome. The main aim of this study is to establish baseline characteristics of patients presenting with breast cancer, which should then inform future efforts to detect breast cancer early in the disease course and improve treatment outcomes.

## Methods

This retrospective chart review evaluated patients who underwent surgical intervention for breast cancer at the Butaro Cancer Center of Excellence (BCCOE) between 2018 and 2023. The expected study population was projected from Operating Room registry data at BCCOE, which indicated an average of five breast cancer surgeries per month (approximately 60 per year); adjusting for holidays and weekends over the five-year study period yielded an expected sample size of approximately 250. Chart review subsequently identified 422 patients who underwent surgical care for breast cancer during this period, from which 300 charts were selected using simple random sampling. This study is part of a larger retrospective chart review and comprehensive evaluation of breast cancer management done in BCCOE. Data extraction utilized both electronic medical records (EMR) and paper-based charts to ensure data completeness. A pretested REDCap electronic data capture tool, validated by clinical experts, was employed for data collection. Extracted data were initially exported to Microsoft Excel for cleaning and subsequently analyzed using IBM SPSS Statistics (version 29). Patient tumor (T), regional lymph node (N), and distant metastasis (M) statuses were recorded independently and aggregated into overall clinical stages according to the American Joint Committee on Cancer (AJCC) staging system (10). This study was designed, executed, and reported in strict accordance with the Strengthening the Reporting of Observational Studies in Epidemiology (STROBE) guidelines for retrospective cross-sectional studies.

### Ethical Consideration

This study received formal ethical approval from the Rwanda National Ethics Committee (RNEC) (IRB number: RNEC/447/2024) and administrative clearance from the institutional review board of Butaro District Hospital (Approval number: 52 BH/EK/DG 2024). Patient confidentiality was maintained throughout the study period by utilizing fully anonymized data extracted from electronic and paper-based health records.

## Results

A total of 422 patients underwent breast surgery at BCCOE from July 2018 to July 2023. We sampled 300 patient charts, which were reviewed using the REDCap tool for retrospective review. The sociodemographic data revealed that the average age of the patients was 50.8 years (SD = 11.8, range = 28 to 82 years). Of the patients, 98% were female, and 2% were male. Additionally, 94% of the patients had health insurance, and 74% were working as farmers. Additional sociodemographic and clinical characteristics as shown (Table 2).

### Risk factor

Among the cases, 65% (N = 195) of the patients were reported to have risk factors (Table 1). Family history accounted for the majority of the non-modifiable risks, which is 5.3% (n = 16). Nulliparity was also a risk factor for 3.7% (n = 11). Among the modifiable risk factors, 55.3% of the cases had a history of alcohol consumption, and the history of tobacco smoking accounted for 21.7% of the cases.

### Eastern Cooperative Oncology Group (ECOG) Performance status

Performance at presentation was also assessed using the ECOG classification. Most patients (81.5%) were found to be in ECOG 0 at diagnosis (Fig. 1). Which means they were fully active and were able to carry out all the pre-disease performance without restriction. Another 16.4% of the cases were in ECOG 1, restricted activity in strenuous exercise but can walk and do light or desk work.

### Comorbidities

The majority (60.3%) of breast cancer patients had at least one comorbidity, while 39.7% had none. Hypertension was the most prevalent comorbidity, affecting approximately one-quarter of patients (25.7%), followed by HIV/AIDS (7.0%). Other comorbid conditions, including chronic lung disease, diabetes, and cardiac disease, were relatively rare, each affecting less than 3% of patients. Overall, the findings indicate that comorbidity is a common finding among breast cancer patients in this cohort, with a predominance of non-communicable conditions, particularly hypertension, alongside a notable burden of HIV/AIDS.

### Tumor staging

Among the total 300 breast cancer patients included in this study, 281 were assigned to be TNM staging, Stage III was the most diagnosed stage, accounting for 38.4% (n = 108) of all cases. Early-stage diagnoses were less frequent, with Stage I comprising 15.3% (n = 43) and Stage II comprising 37.0% (n = 104) of the cases. Stage 0 was rare, seen in only one patient (0.4%). Stage IV was diagnosed in 8.9% of cases (n = 25). When combined, late-stage, Stage III and Stage IV, cases made up nearly half of the total diagnoses (47.3%). This shows a significant burden of late-stage presentation in this patient population.

## Discussion

Timely presentation and accurate clinical assessment at diagnosis are critical for guiding treatment decisions and predicting outcomes in breast cancer care. However, delays in presentation remain a major barrier to achieving optimal outcomes, particularly in resource-limited settings, (11). Understanding tumor characteristics alongside patients’ clinical status, performance level, and sociodemographic profile at the time of diagnosis is essential not only for individual patient management but also for informing targeted public health interventions that promote earlier detection. In this study, we examined the clinical presentation patterns of breast cancer patients in the BCCOE setting. Our study shows that 2% of the breast cancer patients were male, consistent with international reports (12). Most of these women in our study were diagnosed at a relatively younger age compared to other international reports. Median age of diagnosis for our study population was 50.8 years (SD = 11.8, range = 28 to 82 years). Most cancers, including breast cancer, are associated with increased risk with age. The global median age of diagnosis of breast cancer is estimated at 62 years (12). However, studies show the median age in other LMICs is lower. For instance, a study done in Dakar teaching hospital (Senegal) shows that of the 254 patients included in the study, 60.6% were younger than 40 years (13). Similarly, a study done in India assessing the relation of socio-demographic factors with age had a median age of patients at diagnosis of 49 years (14, 15). Early studies in Rwanda also show that mean age at diagnosis was 49.7 years, with the majority (57.2%) diagnosed before age 50 (16).

Our findings are, therefore, consistent with the median age of diagnosis in LMICs rather than the global average. Tylfali et al. highlight increased westernization in LMICs, reflected in shifting lifestyle and reproductive patterns such as delayed childbearing, earlier menarche, and reduced breastfeeding, coupled with the high proportion of young population as possible reasons for the lower median age at diagnosis in LMICs (17) (14). The pattern of evidence in Africa lacks details sufficient to establish a relationship with these factors and age at diagnosis.

Additionally, socioeconomic factors such as profession and education are often evaluated in relation to delays in presentation and receiving care (11, 18, 19). Ibrahim et al, for instance, found that a low level of education was related to greater than 3-month delays in presentation in Nigerian women (20). Our study could not collect educational status due to lack of documentation. However, the routinely recorded data on occupation shows that most patients were farmers (74%). This portion of the Rwandan population tends to live in the rural areas of the country (21).

### Risk Factors

Our study indicates that among the total patients 65% (n = 195) of the patients were reported to have risk factors. Family history was the most common non-modifiable risk factor at 5.3% (n = 16). This figure is comparable to other reported figures on family history of breast cancer which is 5–10% (22). Other important risk factors such as age at early menarche and menopause were not routinely documented on the charts. Systematically capturing these at intake, for example, through a standardized nurse-administered checklist for patients presenting with breast concerns, could help guide future treatment-intensity decisions and is an area for improvement in data collection at this centre.

The other category of risk factors are the modifiable risk factors that are either related to reproductive history such as parity or environmental risk factors such as alcohol consumption or smoking (23). Among these risks, nulliparity was documented as a risk factor in 3.7% of patients (n = 11). Several studies have shown the correlation between nulliparity and rising incidence of breast cancer in Africa (23–25). For instance, a prospective study conducted in Burkina Faso found that the 5.1% of nulliparous women among a cohort of 80 women with breast cancer had 30% higher risk of having breast cancer (24). Reproductive risk factors other than nulliparity such as early menarche and late childbearing and late menopause also have increased the incidence of breast cancer in African countries (25). Our data, collected using retrospective chart review method, was not adequate to establish correlation between breast cancer and reproductive risk factors.

History of alcohol consumption and cigarette smoking has been correlated with several cancer types including breast cancer. A study conducted in Sierra Leone in 2017 found that among a cohort of patients with breast cancer, 14.7% had a history of smoking and 33.6% had a history of alcohol consumption (26). Our study found a relatively higher proportion of smoking and alcohol history among cases, 55.3% of the cases had a history of alcohol consumption, and 21.7% of the cases had history of tobacco smoking. These numbers remain higher when compared to other similar studies conducted in three different sub-Saharan countries that explore the relation between Alcohol and Breast cancer (27). The relation between tobacco, alcohol and cancer has been forecasted to increase in Low- and middle-income countries. A study conducted in 2014 to understand the relation between Tobacco, alcohol and neoplasms in LMICs predicted a 69.9% increase in tobacco-related cancer cases and 68% increase in cancer related to Alcohol in a decade (28). It was assumed that LMICs were in the early stages of the tobacco epidemic 2010s and that cancer cases related to these environmental factors would rise (27). The relatively higher incidence observed in this study compared to previous findings supports this and highlights the need for improved behavioral and environmental measures to control these growing modifiable contributors (26, 27).

### ECOG status

Our study found that ECOG at presentation for most patients (81.5%) was ECOG 0. Another 16.4% of the patients had an ECOG score of 1. Most breast cancer patients usually have a lower ECOG at presentations. For instance, a study examining the association between ECOG classification and clinical outcome reported that 59.8% of breast cancer patients had ECOG- PS classification of categories 0 and 33.6% had ECOG of 1 (8). Our chart review captured ECOG only at initial presentation; post-surgical ECOG was not part of the data as it was not routinely documented. This is a potential area for future follow-up analysis. Kumar et.al evaluating the relationship between ECOG and prognostic factors such as mortality and presentation to the emergency department within 6 months, revealed ECOG can predict mortality and readmission for breast cancer patients. This Suggests ECOG-performance to be an excellent predictor of patient outcome post treatment and for prognostics. This may be because ECOG performance status is more directly linked to disease progression in breast cancer than other symptomatic features, such as malnutrition secondary to dysphagia (9). Monitoring ECOG status in breast cancer patients is therefore important for prognostication and guiding treatment adjustments indicating to a possible area of improvement in following breast cancer patients at BCCOE.

### Tumor staging

The majority of breast cancer cases in East Africa are diagnosed at advanced stages, with Stage III and IV comprising a significant portion of the burden. A recent systematic review by Mulugeta et al. indicates that delayed presentation is a main challenge, with a pooled prevalence of 61.85% (29). Although the study does not provide TNM stage-specific data, it associates delays of over 3 months with more advanced disease at diagnosis, including larger tumors, lymph node involvement, and poorer survival outcomes (29). Consultations with traditional healers, low literacy levels, older age, absence of breast pain, distance to health facilities, and rural residence, were also mentioned as key drivers of late presentation in the study

Country-specific studies in East Africa also show a similar pattern. In Kenya, findings by Ekpa et al. found that Stage III (36%) and Stage IV (32%) represented the majority of cases. Stage II accounted for the remaining 32% (30). It was also noted that patients diagnosed at Stage III progressed to metastasis faster than those diagnosed at Stage II (30, 31). Studies from Uganda had a more pronounced finding with 89.3% of breast cancer patients diagnosed at Stage III or IV, making early-stage diagnosis very rare (31). These figures highlight the systemic challenges in early detection and access to care. Comparing these regional findings to our data, we find it aligns closely with the broader East African trend. In our study, Stage III accounted for 38.8% and Stage IV 8.9%, together representing 47.7% of all late-stage diagnosis cases. While our proportion of Stage IV cases appears slightly lower than in Uganda and Kenya, the high prevalence of Stage III is consistent with regional patterns.

A consistent pattern of late-stage breast cancer presentation is also observed across key Rwandan studies reflecting ongoing systemic barriers and patient-level challenges to early detection (11, 32). In Pace et al.'s earlier study, they reviewed patients who presented with breast mass concerns over 18 months of period in 2015 and found 76% of patients presented with advanced disease, and only 24% were diagnosed at early stage (33). Uyisenga et al. studied on breast cancer staging in Rwanda by analyzing 138 patients diagnosed between January 2015 and December 2018 across three referral hospitals and reported 62.5% of patients presented at Stage III and 7.8% at Stage IV, with only 7.0% diagnosed at Stage I (16). Ntirenganya et al. observed a slightly improved profile, with 45.9% of patients presenting at Stage II, yet 37.1% were still diagnosed at Stage III and 14.1% at Stage IV (32) (see Table below).

Studies attribute delays to factors such as reliance on traditional healers, low education levels, multiple referrals, and geographic barriers (2). The relatively higher proportion of Stage II cases in our cohort (37.0%), or the relative decrease in late-stage diagnosis throughout the years may indicate improved screening programs or improved access over the years (19, 34, 35). In Rwanda, several efforts have been made to improve early detection of breast cancer. These include training community health workers and nurses in breast cancer symptom recognition and clinical breast examinations, integrating breast cancer screening into existing cervical cancer programs, and launching early rural detection programs that increase access to diagnosis and referral (34–36). Others offered clinical services like clinical breast exams and biopsies in district hospitals (35). These efforts to improve early detection may be contributing to the relatively improved distribution of earlier breast cancer diagnosis at BCCOE compared to other hospitals in East Africa. Regardless, nearly half of the cases still present at later stages, emphasising the need to continue strengthening community-level education and other early detection initiatives. Sustaining and scaling these programs, alongside generating local outcomes data to evaluate their impact, represents a concrete next step for Rwanda and other countries in the region to tackle late-stage presentation.

Overall, our findings are consistent with the trend of late-stage breast cancer diagnosis observed across low- and middle-income countries, particularly in sub-Saharan Africa. Rwanda mimics this regional pattern, with a high proportion of patients presenting at advanced stages compared to high-income settings, where early-stage detection is more common. However, recent initiatives aimed at improving early detection have started to yield modest improvements. These efforts suggest a promising direction, but sustained investment and system-wide strengthening are needed to significantly reduce late-stage diagnoses.

## Limitations

This study has several limitations. First, as a retrospective chart review, findings were constrained by the completeness and consistency of clinical documentation. This was particularly discernable in some variables (e.g., menarche/menopause history, educational status). We mitigated this by cross-referencing both electronic and paper-based charts to fill gaps where possible. In similar manner, staging and some risk-factor data were incomplete for a subset of patients (19 of 300 lacked a completely recorded TNM stage); percentages for these variables were therefore calculated using the subset of patients with complete data rather than the full cohort, which should be considered when interpreting these figures.

## Conclusion

The study indicates that breast cancer patients in Rwanda are diagnosed at a relatively younger age compared to the global average. There is also a high prevalence of modifiable risk factors among breast cancer patients. Most patients often present with comorbidities and are diagnosed at a late stage that could potentially complicate treatment outcomes and survival of patients. By characterizing the clinical and sociodemographic profile of patients at presentation, our findings help contextualize the delays in care described in the Introduction and inform strategies to enhance early detection and timely management, Particularly in rural Rwandan settings. Future work should build on these baseline findings to evaluate the impact of ongoing breast cancer early detection and prevention initiatives on patient care and outcome in the country.

## Figures and Tables

**Figure 1:**
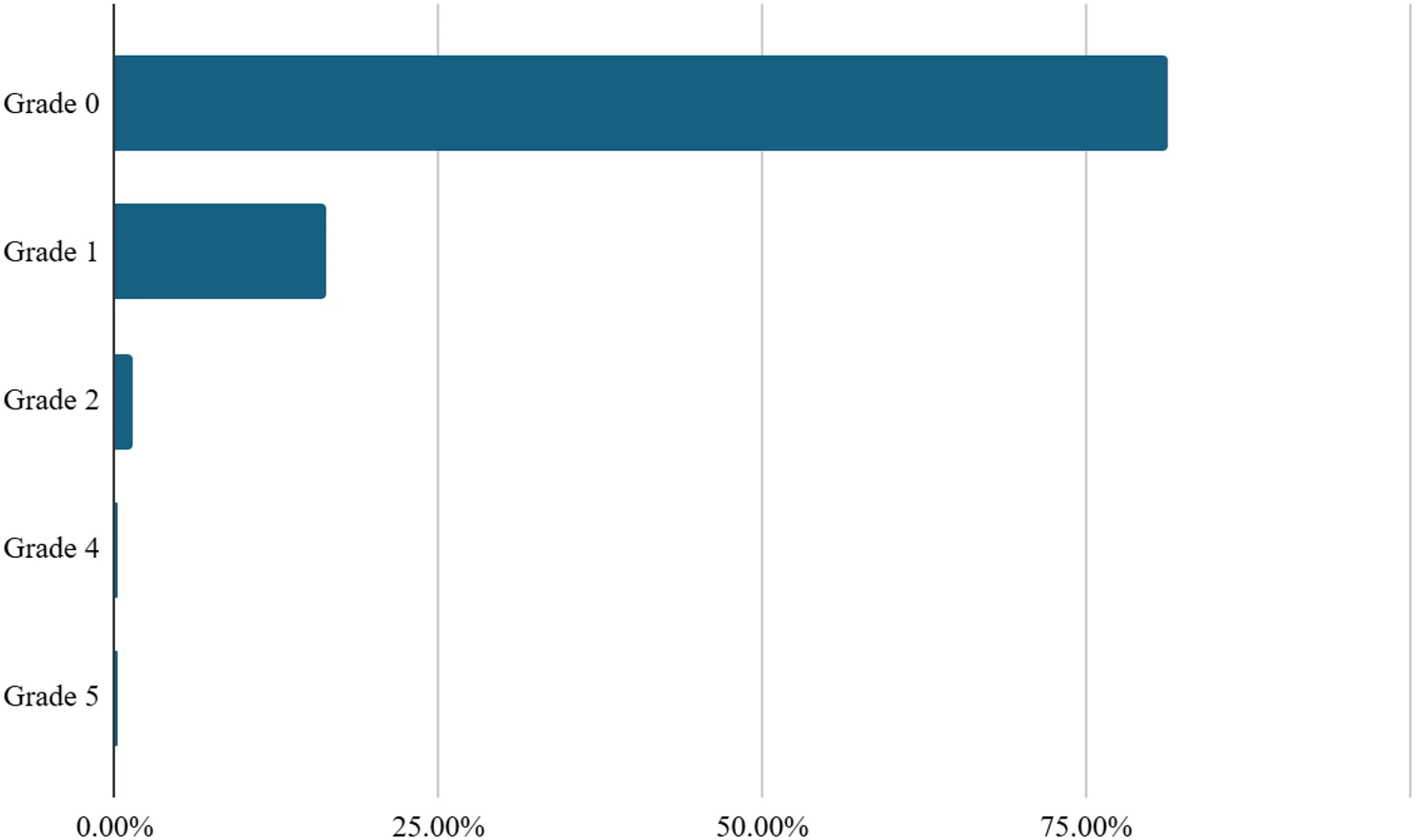
Eastern Cooperative Oncology Group (ECOG) Performance statusdistribution in percentages

**Figure 2:**
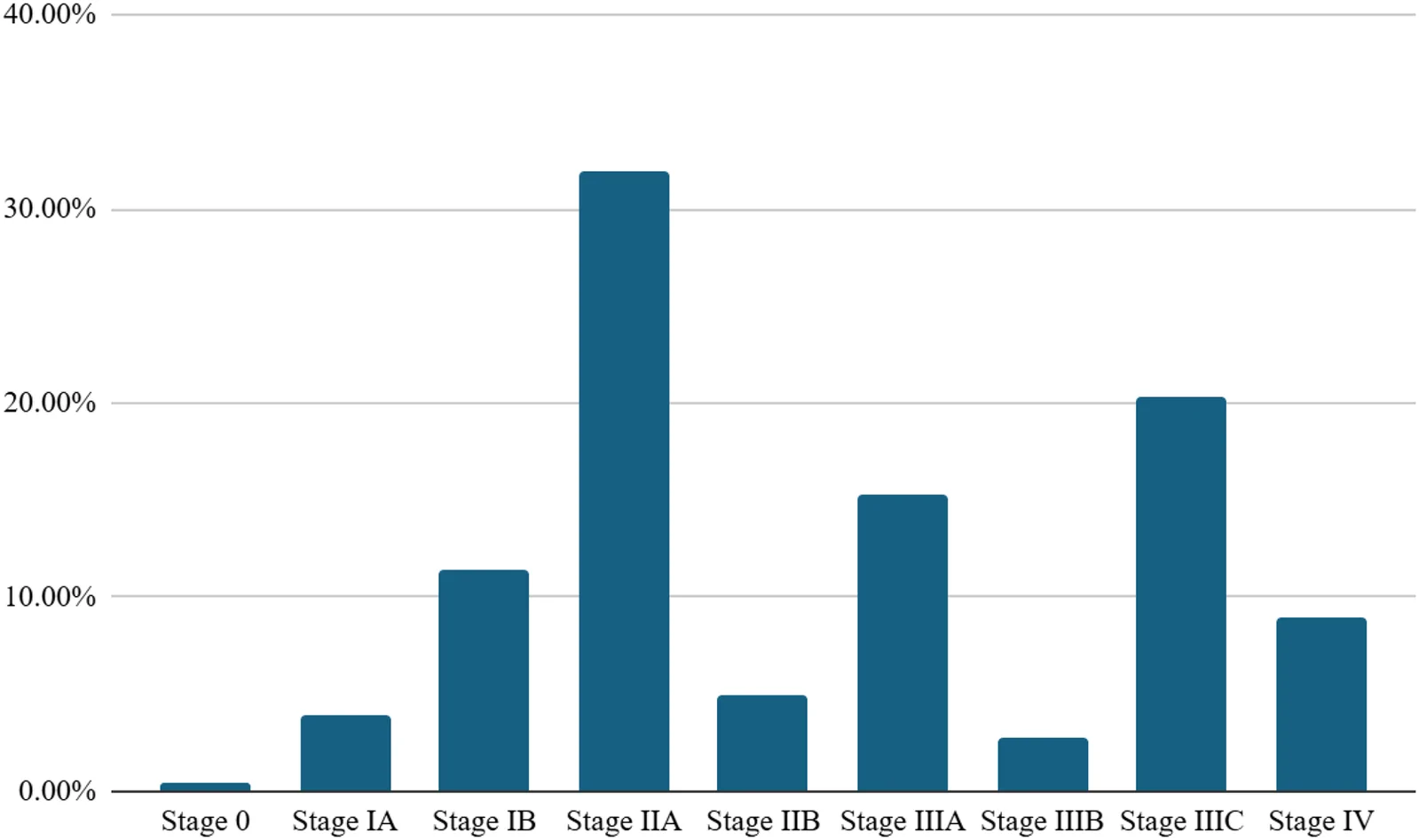
Diagnosed stage at presentation

**Table 1:** Sociodemographic and clinical characteristics of breast cancer patients at Butaro Cancer Center of Excellence (BCCOE) (N = 300)

Characteristic N = 300		n	%
Sex	Female	294	98.0
	Male	6	2.0
Marital status	Married	198	66.0
	Widowed	55	18.3
	Single	19	6.3
	Divorced	8	2.7
	Not recorded	20	6.7
Occupation	Farmer	234	78.0
	Other	28	9.3
	Private employee	11	3.7
	Government employee	13	4.3
	Housewife	7	2.3
	Daily laborer	2	0.7
	Merchant	2	0.7
	Unemployed	2	0.7
	Retired	1	0.3
Risk factors	Any risk factor present	195	65.0
	Alcohol consumption	166	55.3
	Smoking	65	21.7
	Family history	16	5.3
	Nulliparity	11	3.7
	Radiation therapy history	0	0.0
	Not available on chart	12	4.0
Comorbidities	≥ 1 Comorbidity	181	60.3
	No comorbidity	119	39.7
	Hypertension	77	25.7
	HIV/AIDS	21	7.0
	Chronic lung disease	7	2.3
	Diabetes	1	0.3
	Cardiac disease	1	0.3

**Table 2:** TNM Staging breast cancer patients at Butaro Cancer Center of Excellence (BCCOE) (N = 300)

TNM Stage	n	%
Stage 0	1	0.4
Stage IA	11	3.9
Stage IB	32	11.4
Stage I (subtotal)	43	15.3
Stage IIA	90	32.0
Stage IIB	14	5.0
Stage II (subtotal)	104	37.0
Stage IIIA	43	15.3
Stage IIIB	8	2.8
Stage IIIC	57	20.3
Stage III (subtotal)	108	38.4
Stage IV	25	8.9
Total staged	281	100.0

**Table 3:** Tumor Stage Distribution Across Rwandan Breast Cancer Studies

Tumor Stage	Uyisenga (2020)	Ntirenganya (2024)	Pace (2015)	Our Findings
**Stage I**	7.0%	2.9%	–	6.1%
**Stage II**	24.8%	45.9%	24% (I + II)	37.0%
**Stage III**	62.5%	37.1%	52%	38.4%
**Stage IV**	7.8%	14.1%	24%	8.9%
**Late-Stage (III + IV)**	70.3%	47.9%	76%	47.3%

## Data Availability

All data that was used for this manuscript is available upon reasonable request.
